# Whole-Genome Sequencing of Macrolide-Resistant *Mycoplasma pneumoniae* Strain S355, Isolated in China

**DOI:** 10.1128/genomeA.00087-16

**Published:** 2016-03-17

**Authors:** Shaoli Li, Fei Liu, Hongmei Sun, Baoli Zhu, Na Lv, Guanhua Xue

**Affiliations:** a Capital Institute of Pediatrics, Beijing, China; b Institute of Microbiology, Chinese Academy of Sciences, Beijing, China

## Abstract

Macrolide-resistant *Mycoplasma pneumoniae* plays an important role in refractory *M. pneumoniae* pneumonia. Here, we present the whole-genome sequencing of the macrolide-resistant *M. pneumoniae* strain S355. The annotated full-genome sequence might provide a new insight into drug resistance in *M. pneumoniae* and can help pediatricians recognize the disease earlier.

## GENOME ANNOUNCEMENT

Macrolide-resistant *Mycoplasma pneumoniae* (MRMP) strains are a common cause of refractory *M. pneumoniae* pneumonia (1). The mutations on the 23S rRNA gene were thought to be responsible for the drug resistance in *M. pneumoniae* (2). The rates of MRMP have been up to >90% in Asia (3, 4). Here, we present a genome sequence of MRMP strain S355.

S355 was isolated in China. Next, it was cultured in pleuropneumonia-like organism medium with some extra nutrients at 37°C for several days. The bacterial suspension was harvested, and genomic DNA was extracted using the QIAamp Mini DNA kit (Qiagen, Hilden, Germany), according to the manufacturer’s instructions, and the DNA was sonicated using a Diagenode bioruptor (Diagenode SA, Liège, Belgium).

The Illumina HiSeq 2000 sequencing platform was used. A total of 633,894 paired-end reads with an average read length of 100 bp, corresponding to 150-fold coverage of the genome, were generated. Raw reads were first filtered using the DynamicTrim and LengthSort Perl scripts provided in the SolexaQA suite and then assembled using SOAP*denovo* (http://soap.genomics.org.cn), yielding 15 scaffolds with a mean length of 53,549 bp. Gaps were closed by PCR, and subsequently, the ABI-3730 genetic analyzer (Applied Biosystems, CA) was used. The genome sequence was annotated using the NCBI Prokaryotic Genome Annotation Pipeline (NCBI_PGAP). The complete genome comprises 801,203 bp of chromosomal DNA (39.9% G+C content), containing 694 coding sequences (CDSs), 1 rRNA operon, and 37 tRNAs. SOAPsnp was used to score single nucleotide polymorphisms (SNPs) from the aligned reads (5). The short reads were first aligned onto the M129 reference genome using the SOAP2 program. In total, 352 SNPs were identified.

In summary, the genome sequence reported here provides a full understanding of the gene mutations of MRMP.

### Nucleotide sequence accession number.

This whole-genome shotgun project has been deposited in GenBank under the accession no. CP013829. The version described in this paper is the first version.
